# Evidence for two candidate tumour suppressor loci on chromosome 9q in transitional cell carcinoma (TCC) of the bladder but no homozygous deletions in bladder tumour cell lines

**DOI:** 10.1038/sj.bjc.6690383

**Published:** 1999-05-01

**Authors:** A A G van Tilborg, L E Groenfeld, T H van der Kwast, E C Zwarthoff

**Keywords:** bladder cancer, transitional cell carcinoma, chromosome 9, deletion mapping, tumour suppressor genes, loss of heterozygosity

## Abstract

The most frequent genetic alterations in transitional cell carcinoma (TCC) of the bladder involve loss of heterozygosity (LOH) on chromosome 9p and 9q. The LOH on chromosome 9p most likely targets the *CDKN2* locus, which is inactivated in about 50% of TCCs. Candidate genes that are the target for LOH on chromosome 9q have yet to be identified. To narrow the localization of one or more putative tumour suppressor genes on this chromosome that play a role in TCC of the bladder, we examined 59 tumours with a panel of microsatellite markers along the chromosome. LOH was observed in 26 (44%) tumours. We present evidence for two different loci on the long arm of chromosome 9 where potential tumour suppressor genes are expected. These loci are delineated by interstitial deletions in two bladder tumours. Our results confirm the results of others and contribute to a further reduction of the size of these regions, which we called TCC1 and TCC2. These regions were examined for homozygous deletions with EST and STS markers. No homozygous deletions were observed in 17 different bladder tumour cell lines. © 1999 Cancer Research Campaign


*British Journal of Cancer* (1999) **80**(3/4), 489–494
© 1999 Cancer Research Campaign
Article no. bjoc.1998.0383

### Evidence for two candidate tumour suppressor loci on chromosome 9q in transitional cell carcinoma (TCC) of the bladder but no homozygous deletions in bladder tumour cell lines

**AAG van Tilborg, LE Groenfeld, TH van der Kwast and EC Zwarthoff**

**Summary** The most frequent genetic alterations in transitional cell carcinoma (TCC) of the bladder involve loss of heterozygosity (LOH) on chromosome 9p and 9q. The LOH on chromosome 9p most likely targets the *CDKN2* locus, which is inactivated in about 50% of TCCs. Candidate genes that are the target for LOH on chromosome 9q have yet to be identified. To narrow the localization of one or more putative tumour suppressor genes on this chromosome that play a role in TCC of the bladder, we examined 59 tumours with a panel of microsatellite markers along the chromosome. LOH was observed in 26 (44%) tumours. We present evidence for two different loci on the long arm of chromosome 9 where potential tumour suppressor genes are expected. These loci are delineated by interstitial deletions in two bladder tumours. Our results confirm the results of others and contribute to a further reduction of the size of these regions, which we called TCC1 and TCC2. These regions were examined for homozygous deletions with EST and STS markers. No homozygous deletions were observed in 17 different bladder tumour cell lines.

**Keywords:** bladder cancer; transitional cell carcinoma; chromosome 9; deletion mapping; tumour suppressor genes; loss of heterozygosity

Bladder cancer is the fifth most common cancer in males. Over 95% of all bladder cancers in industrialized countries are transitional cell carcinomas (TCCs). TCCs are presented in two ways: superficial papillary tumours, confined to the mucosa and lamina propria; and invasive tumours spreading beyond the lamina propria into detrusor muscle. The remaining 5% of tumours include squamous cell carcinomas, adenocarcinomas and carcinoma in situ.

Frequent somatic allelic loss is regarded as a hallmark of tumour suppressor gene (TSG) inactivation. In TCCs, cytogenetic studies and loss of heterozygosity (LOH) analyses have revealed a number of chromosomal aberrations, including deletion of chromosome 9p and/or 9q (Devlin et al, 1994) (Keen and Knowles, 1994) (Cairns et al, 1993; Linnenbach et al, 1993; Orlow et al, 1994), and deletions of chromosome 11p (Shipman et al, 1993), 18q (Brewster et al, 1994), chromosome 8 (Knowles et al, 1993; Takle and Knowles, 1996), 4p (Elder et al, 1994; Polascik et al, 1995) and 14q (Chang, 1995). LOH of markers on chromosome 9 is found in TCCs of all grades and stages, suggesting that the inactivation of a putative TSG on this chromosome is an early event in the development of bladder cancer. Several groups (Ruppert et al, 1993; Habuchi et al, 1995; Simoneau et al, 1996) reported evidence for the presence of more than one TSG that can contribute to the development of bladder cancer on chromosome 9. The *CDKN2A* (*p16, MTS1*) and *CDKN2B* (*p15*) genes are localized on the short arm of chromosome 9. Recent studies showed the inactivation of these genes in as much as 40–50% of bladder tumours (Cairns, 1995; Akao et al, 1997). More detailed deletion mapping on the long arm revealed two regions of loss (Habuchi et al, 1995; Simoneau et al, 1996). Small interstitial deletions covering the location of the marker D9S195 were recently reported in five TCCs. The shortest region of overlap of these deletions is estimated to amount to about 840 kb. The putative TSG in this deleted in bladder cancer region (DBC1) was called DBCCR1 (Habuchi et al, 1998). This DBC1 region does not overlap with the two other regions described by Habuchi et al (1995) and Simoneau et al (1996). Thus, the combined data provide evidence for three TSGs on chromosome 9q that may play a role in the pathogenesis of bladder cancer.

In the present study we used a polymerase chain reaction (PCR)-based microsatellite assay to further delineate the extent of the deletions at chromosome 9q. A combination of our data with those of others, supports the view that apart from the DBC1 region two other putative TSG loci may exist on chromosome 9q. These two regions were called TCC1 and TCC2. We screened 17 bladder tumour cell lines for homozygous deletions in these areas. No evidence for homozygous deletions was obtained.

### MATERIALS AND METHODS

### DNA preparation

Matched pairs of 59 paraffin-embedded bladder tumours and normal control tissue of the same patient were selected. Paraffin sections were examined microscopically by a pathologist (Th v/d K). Parts that represented tumour tissue were punched out of the original paraffin blocks and newly embedded. DNA was isolated by proteinase K (2 mg ml$^{-1}$) digestion of deparaffinized 5-μm

*Received 1 June 1998*
*Revised 9 November 1998*
*Accepted 24 November 1998*

*Correspondence to:* EC Zwarthoff, Department of Pathology, Erasmus University, PO Box 1738, 3000 DR Rotterdam, The Netherlands

**Table 1** Percentages of allele signal left for bladder tumours tcc36 and tcc39 as determined with the Phosphorimager[a]

| Marker | tcc36 | tcc39 |
|---|---|---|
| D9S165 | | 0.94 |
| D9S166 | | 0.34 |
| D9S264 | | 0.15 |
| D9S283 | 0.87 | 0.13 |
| D9S197 | 0.51 | |
| D9S280 | | 0.19 |
| D9S287 | 0.57 | |
| D9S180 | 0.59 | 0.22 |
| D9S272 | | NM[c] |
| D9S1783 | | NM |
| D9S176 | | 0.77 |
| D9S173 | | 0.89 |
| D9S177 | | 1.14 |
| D9S154 | 0.64 | |
| D9S275 | 0.73 | |
| D9S195 | 0.37 | |
| D9S179 | 0.38 | 0.76 |
| D9S164 | 0.44 | |
| D9S1818 | NI[b] | |
| D9S1826 | 0.60 | |
| D9S158 | 0.86 | 0.60 |
| D9S1838 | 0.65 | |

[a]The percentages were determined by comparing the intensity of the lost allele in the tumour with the intensity of the same allele in normal control DNA, in relation to the intensity of the other retained allele. [b]NI, not informative; [c]NM, not measured.

**Table 2** Overview of the ESTs and STSs used for HD mapping, ordered from centromere to telomere

| | Located in TCC1 | Located in TCC2 |
|---|---|---|
| 1 stSG8675 | 43 A002D08 | 84 U18543 |
| 2 WI-30336 | 44 A008R29 | 85 IB3089 |
| 3 A002Y36 | 45 IB543 | 86 WI-13592 |
| 4 WI-11585 | 46 WI-2958 | 87 WI-11542 |
| 5 CKS2 | 47 WI-6937 | 88 WI-6257 |
| 6 WI-11909 | 48 IR10 | 89 WI-12734 |
| 7 WI-16825 | 49 WI-13546 | 90 ROP |
| 8 WI-12646 | 50 PTCH | 91 WI-11957 |
| 9 stSG2370 | 51 WI-14826 | 92 FB23F1 |
| 10 stSG8105 | 52 WI-1941 | 93 WI-15097 |
| 11 WI-2414 | 53 WI-2013 | 94 WI-13608 |
| 12 stSG9248 | 54 stSG9221 | 95 NIB1929 |
| 13 WI-4860 | 55 WI-9350 | 96 WI-14271 |
| 14 A004T01 | 56 A006115 | 97 WI-11577 |
| 15 IB2336 | 57 WI-6378 | 98 WI-12991 |
| 16 IB3559 | 58 stSG8121 | |
| 17 WI-9447 | 59 WI-9840 | |
| 18 WI-2331 | 60 WI-9914 | |
| 19 WI-6758 | 61 WI-9212 | |
| 20 WI-17567 | 62 A003P31 | |
| 21 WI-9905 | 63 WI-7974 | |
| 22 A006N11 | 64 WI-7285 | |
| 23 A006U15 | 65 WI-11414 | |
| 24 NIB973 | 66 A001T44 | |
| 25 stSG1471 | 67 WI-8684 | |
| 26 stSG2118 | 68 TGFBR1 | |
| 27 NIB722 | 69 A008N47 | |
| 28 stSG2205 | 70 A005N10 | |
| 29 stSG3724 | 71 WI-7447 | |
| 30 WI-7541 | 72 WI-5249 | |
| 31 WI-13139 | 73 WI-14669 | |
| 32 A007K29 | 74 WI-7344 | |
| 33 WI-6338 | 75 WI-3790 | |
| 34 WI-6428 | 76 NIB1437 | |
| 35 WI-4577 | 77 WI-15742 | |
| 36 WI-532 | 78 WI-688 | |
| 37 WI-8025 | 79 SGC31311 | |
| 38 stSG1737 | 80 WI-11370 | |
| 39 stSG2403 | 81 WI-2008 | |
| 40 WI-15517 | 82 A008T08 | |
| 41 A001U11 | 83 WI-4017 | |
| 42 WI-2820 | | |

sections, followed by phenol–chloroform extraction and ethanol precipitation. A haematoxylin and eosin stain of sections flanking the sections used for DNA isolation was again controlled by the pathologist. In general, the percentage tumour tissue in the material dissected by this procedure was estimated to be over 90%.

The following bladder tumour cell lines were used: 253J, 575A, 647V, 1207, 5637, J82, Jon, RT4, RT112, SCaBER, SD, SW780, SW800, SW1710, T24, VMCubI and VMCubII. Dr D Chopin, Paris, kindly provided the cell lines 1207 and 647V. Genomic DNA was prepared according to standard procedures (Sambrook et al, 1989).

### LOH analysis

For LOH analysis, microsatellite primer sequences were obtained from the Genome DataBase (http://gdbwww.gdb.org/gdb). Thirty primer pairs were used. On the short arm, the markers D9S178, D9S171, D9S168, D9S165 and D9S156 were included. On the long arm, the markers D9S153, D9S154, D9S158, D9S164, D9S166, D9S173, D9S176, D9S177, D9S179, D9S180, D9S195, D9S196, D9S197, D9S257, D9S264, D9S272, D9S275, D9S278, D9S280, D9S283, D9S287, D9S1783, D9S1818, D9S1826 and D9S1838 were used. Template DNA (50 ng) was amplified in a total volume of 15 µl reaction mixture containing 2.5 mM dNTPs, 10 pmol of the appropriate primer combination and 0.25 units of *Taq* polymerase (Supertaq). Products were labelled with $\alpha$-$^{32}$P-dATP.

Thermal cycling consisted of initial denaturation at 95°C for 5 min, followed by 32 cycles of each 55°C for 45 s, 72°C for 40 s and 94°C for 40 s. The final elongation step was 72°C for 10 min. PCR products were separated on 6% denaturing polyacrylamide gels. Detection was done by autoradiography and, when necessary, followed by quantification using a Phosphorimager (Molecular Dynamics, Sunnyvale, CA, USA). An allele was considered to be lost when the intensity of the remaining signal was less than 50% compared to the signal of the same allele in the matching control DNA of the same patient.

### Homozygous deletion screening

For the homozygous deletion mapping, 98 primer sequences were obtained from the Whitehead Institute (http://www-genome.wi.mit.edu/), the Sanger Centre (http://www.sanger.ac.uk) and The Institute of Genome Research (TIGR) (http://www.tigr.org). All primer sequences were from sequence tagged sites (STS) or expressed sequence tags (EST) mapped between our TCC1 and TCC2 border markers (http://www.ncbi.nlm.nih.gov/Science96). Amplification was done as described for the LOH analysis, with the exception of the presence of a second control primer set in the reaction mixture. As a control, primers were used for the NF2 (exon 5 and 11; Jacoby et al, 1994) or MN1 genes on chromosome 22 (bp 5304–5421, forward: MN1–16, 5′-AGG TTG GTA CCT

 © Cancer Research Campaign 1999

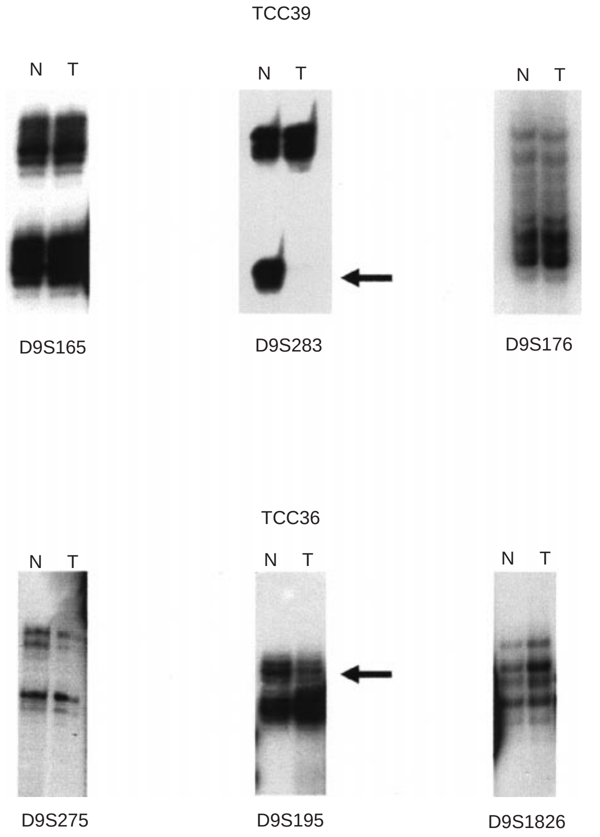

**Figure 1** Autoradiographs illustrating the LOH analyses for tcc36 and tcc39. N: matched control DNA; T: tumour DNA. Arrows indicate deleted alleles. (top) tcc39: markers D9S165 and D9S176 show retention, while marker D9S283 shows loss of the lower allele. (bottom) tcc36: markers D9S275 and D9S1826 show retention, while marker D9S195 shows a lower intensity of the upper allele

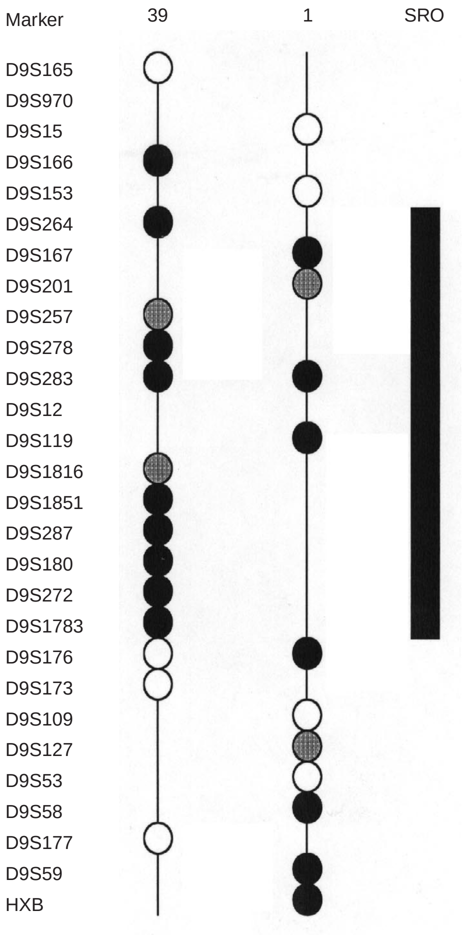

**Figure 2** The borders of the TCC1 region. Markers are shown in linkage and physical mapping order according to the Whitehead Institute contig data and CEPH/Généthon data. (○) retention; (●) loss of heterozygosity; (◍) not informative. On the right side, a vertical bar indicates the potential smallest region of overlap, based on our results with tcc39 and the results of Habuchi et al (1995)

GCT TAG TG, reverse: MN1–13, 5′-GGG TTA ACA CTG GTA ACA TAC), since there are no data suggesting the involvement of either of these genes or the chromosome in bladder cancer. Since the presence of a homozygous deletion in the *CDKN2A* gene was known in eight of the 17 cell lines used, primers were included for a 167 bp product spanning an intron–exon boundary of the *CDKN2A* gene (Nobori et al, 1994). The detection of these deletions was used as a positive control.

### RESULTS

### LOH analysis

Fifty-nine bladder tumours were screened for LOH of markers on chromosome 9. Twenty-six tumours (44%) showed LOH for one or more markers. No microsatellite instability was seen. Of these, two tumours had a deletion confined to the p arm, in ten the loss was confined to the q arm and in 12 cases both p and q arms were affected. Losses on the short arm overlap the region containing the *CDKN2* locus, which is located telomeric to marker D9S171. Two individual tumours were found to obtain different interstitial deletions on chromosome 9q, suggesting two different TSG loci on this chromosome arm. These are discussed in detail in the following sections. Other regions of loss that were observed on 9q could target both putative TSG loci on 9q and/or the *CDKN2* locus and did not contribute to a further delineation of these loci.

### An interstitial deletion between D9S165 and D9S176

In tcc39 an interstitial deletion was observed between the flanking markers D9S165 and D9S176. No loss was observed for three microsatellite markers on 9p. Examples of the LOH analysis of 9q are shown in Figure 1A. Between D9S165 and D9S176, a clear LOH was observed for seven microsatellites. The autoradiogram for one of these, D9S283, is shown in Figure 1A. The extent of loss was also calculated with the Phosphorimager. The results obtained are depicted in Table 1 in the lane marked tcc39. For D9S283, the signal of the lost allele was measured to be 13% of the control allele from normal tissue. For some of the other markers, slightly higher values were observed. This is probably due to the fact that when two alleles are relatively close together and comprise several stutter bands, they contribute to each other's background. In some cases, i.e. for D9S272 and D9S1783, this makes the quantitative analysis impossible, although with the eye a clear LOH is evident. The region deleted in tcc39 is approximately 48 cM in size. Tcc39 was classified by the pathologist as Ta/grade 2.

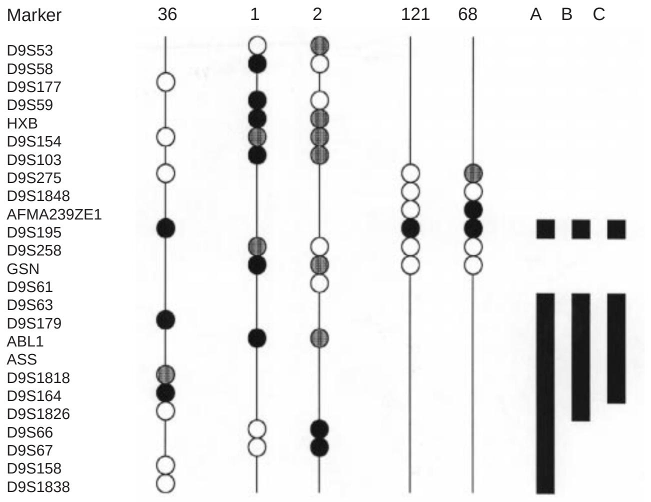

**Figure 3** The borders of the DBC1 and TCC2 regions. Markers are shown in linkage and physical mapping order according to the Whitehead Institute contig data and CEPH/Généthon data. (○) retention; (●) loss of heterozygosity; (◍) not informative. On the right side, vertical bars indicate the possible smallest regions of overlap, based on our results with tcc36 and the results of Habuchi et al (1995, 1997) and Simoneau et al (1996). For an explanation of the three possible SROs for TCC2 (A, B, and C), see text

### An interstitial deletion between D9S275 and D9S1826

Tcc36 is a T1/grade 2 bladder tumour in which 40% of the cells are monosomic for chromosome 9 as determined by in situ hybridization using the chromosome 9 heterochromatin region probe pHUR98 (van Tilborg et al, 1998). Thus, tcc36 is heterogeneous with respect to its genomic constitution. This partial loss of one copy of chromosome 9 is also observed in the LOH analyses and is reflected by the measured intensities of allele signals of around 60% as shown in Table 1. Three microsatellites show a remaining signal of approximately 30–40% when compared to the control DNA. This most likely reflects an interstitial deletion of 31 cM flanked by markers D9S275 and D9S1826. Figure 1B shows representative autoradiograms of the LOH analyses of tcc36. Based on the signal intensities as measured by the Phosphorimager, the most plausible model to explain these findings would be that one allele of a putative TSG which is located within the interstitially deleted area was first inactivated and that two individual second hits targeting the other allele occurred in separate cells: (a) loss of an entire copy of chromosome 9 as reflected by the subpopulation of 40% of the tumour cells that are monosomic for chromosome 9 and (b) an interstitial deletion of the same copy of chromosome 9 present in approximately 25% of the tumour cells.

### Homozygous deletion analysis

We next screened DNA from 17 bladder tumour cell lines for homozygous deletions in these areas. For the more centromeric region, 83 ESTs and STSs were selected between the markers D9S153 and D9S176, a region of 27 cM. The borders of this region are indicated in Figure 2, they were deduced based on our results with tcc39 and the results of Habuchi et al (1995). The markers for the homozygous deletion analysis are listed in Table 2. When the markers are randomly distributed, this results in a density of one marker per 300 kb. Special attention was paid to the prevention of contaminating the PCR-reaction to avoid false positives, by strictly separating the equipment used to handle amplified DNA from other equipment. Separate work areas were used for pre- and post-amplification steps. Random negative controls were included. No homozygous deletions were found. In addition, the region with an interstitial deletion as defined by tcc36 was screened with 15 sets of PCR primers, representing a density of 1 marker per 500 kb. However, no evidence for homozygous deletions was obtained. As a control we also screened the cell lines for deletions of the *CDKN2* locus. Deletions were observed in eight of the 17 cell lines tested. This confirms data obtained by others (Southgate et al, 1995; Williamson et al, 1995; Tamimi et al, 1996).

### DISCUSSION

The purpose of this study was to further define chromosome 9q deletions in TCCs. Previous LOH analyses predicted that 57% of tumours had deletions on chromosome 9p and 9q (Habuchi et al, 1995). The putative presence of two or more TSGs on the same chromosome complicates the interpretation of the LOH analysis. Most estimations for the losses of the whole chromosome are based on allelotyping studies in which a limited number of 9p and 9q markers were used. This causes high percentages of apparent complete loss of chromosome 9. It is our experience that when more markers are used most of these apparent cases of monosomy are in fact large terminal or interstitial deletions. This emphasizes the importance of testing as many informative polymorphic markers as possible. The *CDKN2A (p16)*/*CDKN2B (p15)* tumour suppressor genes are located on the short arm of chromosome 9. Loss of one or even both copies of these genes was shown to occur in at least 40–50% of TCCs (Cairns, 1995; Akao et al, 1997). In some cases, the region of LOH spreads from the *CDKN2* region beyond the centromere into the q arm of chromosome 9. Such a deletion could target the *CDKN2* region, a locus on the q arm or even both. In addition, losses that are confined to the q arm of chromosome 9 suggest the existence of more than one candidate bladder gene on this arm. Also here it is often not possible to define to which region the observed LOH contributes.

Our LOH results confirm the hypothesis that there are at least two different putative tumour suppressor gene loci on the q arm. The first, more centromeric region is called TCC1. The borders of this new region, as depicted in Figure 2, are defined by the interstitial deletion in tcc39 as reported in this work and an interstitial deletion in a bladder tumour number 1 as published by Habuchi et al (1995). For the definition of the region we have excluded tumours in which LOH was observed based on only one tested marker. Our results place the lower border of the TCC1 region at marker D9S176, instead of D9S109. This reduces the size of the region by 6 cM from 33 to 27 cM. In both our case tcc39 and case 1, the signal intensities of the remaining alleles are very low, suggesting that the gene targeted by these deletions is inactivated in most, if not all, tumour cells. Thus, inactivation of this gene may represent an early event in the pathogenesis of these tumours.

For the definition of a second region, several possibilities exist. These are shown in Figure 3. In this Figure the interstitial deletion in tcc36 (this paper) is shown next to deletions as published by the group of Knowles (Habuchi et al, 1995, 1997). The DBC1 region was deducted from short interstitial deletions in five separate tumours that have a shortest region of overlap of 840 kb in which marker D9S195 is located (Habuchi et al, 1997). The first conclusion from these combined data is that it is impossible to attribute

 *© Cancer Research Campaign 1999*

all deletions to one region. For instance, tumours 36 and 1 can both target the DBC1 region, but case 2 clearly falls outside this region. The three different possible SRO regions based on these data are also shown in Figure 3. In Figure 3 lane A, it is assumed that both tcc36 and case 1 target the DBC1 region. This would result in a TCC2 region defined by case 2. In option B, tcc36 targets DBC1 and case 1 the TCC2 region, and reversely in option C, the case 2 deletion targets DBC1 and tcc36 TCC2. As a result, the size of the TCC2 region can vary from 30 to 40 cM.

Approximately 40% of the cells in tcc36 are monosomic for chromosome 9 and in an additional 25% an interstitial deletion of the same copy of chromosome 9 occurred. This suggests that the interstitial deletion and the loss of an entire chromosome may target the same TSG and that for this gene these two events represent separate second hits. These findings suggest that the inactivation of the proposed TSG at this location may not have been one of the first hits in the pathogenesis of tcc36.

Losses of chromosome 9q have also been observed in basal cell carcinoma (Shanley et al, 1995), squamous cell carcinoma of the head and neck (Ah-See et al, 1994), oesophagus carcinoma (Miura et al, 1996), ovarian cancer (Shultz et al, 1995), renal cell carcinoma (Cairns et al, 1995) and small-cell lung cancer (Merlo et al, 1994). Since the SROs for bladder cancer are still very large, with the exception of the DBC1 region, it cannot be excluded that the losses seen in other tumours target the same TSGs. Recently, the gene responsible for sporadic basal cell carcinoma of the skin and the hereditary disorder NBCCS was identified. This *PTCH* gene is located within the TCC1 region. However, no mutations in this gene in bladder tumours were observed (Simoneau et al, 1996; our unpublished results). For oesophagus carcinoma, the region containing a putative TSG has been narrowed down to about 200 kb, between the markers D9S155 and D9S177. These microsatellites are positioned distal of the TCC1 region and proximal to the DBC1 region. In ovarian cancer, LOH is found around the gelsolin gene (GSN), where the DBC1 gene is expected.

Both the TCC1 and TCC2 regions were screened for the presence of homozygous deletion in 17 bladder tumour cell lines with in total 100 microsatellite markers, with an average spacing of 300–500 kb. Deletions of the *CDKN2* region are often between 50 and 500 kb or more in size (Williamson et al, 1995). Other homozygous deletions vary between 130 kb in B-cell chronic lymphocytic leukaemia (Corcoran et al, 1998), and 3 Mb in non-small cell lung cancer (Kohno et al, 1998). This suggests that the homozygous deletion targeting the TCC1 and TCC2 loci are either much smaller in size than those observed for other loci or that the putative TSGs are not readily inactivated by homozygous deletions.

Several interesting genes in the TCC2 region are known: the transforming growth factor β receptor type I gene (TGFBR1), the death associated protein kinase 1 gene (DAPK1) and the tuberous sclerosis 1 gene (TSC1). Further studies to identify the genes involved in bladder cancer should include these genes as candidates.

### REFERENCES

Ah-See KW, Cooke TG, Pickford IR, Soutar D and Balmain A (1994) An allelotype of squamous carcinoma of the head and neck using microsatellite markers. *Cancer Res* **54**: 1617–1621

Akao T, Kakehi Y, Itoh N, Ozdemir E, Shimizu T, Tachibana A, Sasaki MS and Yoshida O (1997) A high prevalence of functional inactivation by methylation modification of p16$^{INK4A}$/CDKN2/ MTS1 gene in primary urothelial cancers. *Jpn J Cancer Res* **88**: 1078–1086

Brewster SF, Gingell JC, Browne S and Brown KW (1994) Loss of heterozygosity on chromosome 18q is associated with muscle-invasive transitional cell carcinoma of the bladder. *Br J Cancer* **70**: 697–700

Cairns P (1995) Frequency of homozygous deletion at p16/CDKN2 in primary human tumours. *Nat Genet* **11**: 210–212

Cairns P, Shaw ME and Knowles MA (1993) Initiation of bladder cancer may involve deletion of a tumour-suppressor gene on chromosome 9. *Oncogene* **8**: 1083–1085

Cairns P, Tokino K, Eby Y and Sidransky D (1995) Localization of tumour suppressor loci on chromosome 9 in primary human renal cell carcinomas. *Cancer Res* **55**: 224–227

Chang WYH (1995) Novel suppressor loci on chromosome 14q in primary bladder cancer. *Cancer Res* **55**: 3246–3249

Corcoran MM, Rasool O, Liu Y, Iyengar A, Grander D, Ibbotson RE, Merup M, Wu X, Brodyansky V, Gardiner AC, Juliusson G, Chapman RM, Ivanova G, Tiller M, Gahrton G, Yankovsky N, Zabarovsky E, Oscier DG and Einhorn S (1998) Detailed molecular delineation of 13q14.3 loss in B-cell chronic lymphocytic leukemia. *Blood* **91**: 1382–1390

Devlin J, Keen AJ and Knowles MA (1994) Homozygous deletion mapping at 9p21 in bladder carcinoma defines a critical region within 2 cM of IFN-A. *Oncogene* **9**: 2757–2760

Elder PA, Bell SM and Knowles MA (1994) Deletion of two regions on chromosome 4 in bladder carcinoma: definition of a critical 750 kb region at 4p16.3. *Oncogene* **9**: 3433–3436

Habuchi T, Devlin J, Elder PA and Knowles MA (1995) Detailed deletion mapping of chromosome 9q in bladder cancer: evidence for two tumour suppressor loci. *Oncogene* **11**: 1671–1674

Habuchi T, Yoshida O and Knowles MA (1997) A novel candidate tumour suppressor locus at 9q32–33 in bladder cancer: localization of the candidate region within a single 840 kb YAC. *Hum Mol Genet* **6**: 913–919

Habuchi T, Luscombe M, Elder PA and Knowles MA (1998) Structure and methylation-based silencing of a gene (DBCCR1) within a candidate bladder cancer tumor suppressor region at 9q32–33. *Genomics* **48**: 277–288

Jacoby LB, MacCollin M, Louis DN, Mohney T, Rubio MP, Pulaski K, Trofatter JA, Kley N, Seizinger B, Ramesh V and Gusella JF (1994) Exon scanning for mutation of the NF2 gene in schwannomas. *Hum Mol Genet* **3**: 413–419

Keen AJ and Knowles MA (1994) Definition of two regions of deletion on chromosome 9 in carcinoma of the bladder. *Oncogene* **9**: 2083–2088

Knowles MA, Shaw ME and Proctor AJ (1993) Deletion mapping of chromosome 8 in cancers of the urinary bladder using restriction fragment length polymorphisms and microsatellite polymorphisms. *Oncogene* **8**: 1357–1364

Kohno T, Kawanishi M, Matsuda S, Ichikawa H, Takada M, Ohki M, Yamamoto T and Yokota J (1998) Homozygous deletion and frequent allelic loss of the 21q11.1–q21.1 region including the ANA gene in human lung carcinoma. *Genes Chromosomes Cancer* **21**: 236–243

Linnenbach AJ, Pressler LB, Seng BA, Kimmel BS, Tomaszewski JW and Malkowicz SB (1993) Characterization of chromosome 9 deletions in transitional cell carcinoma by microsatellite assay. *Hum Mol Genet* **2**: 1407–1411

Merlo A, Gabrielson E, Mabry M, Vollmer R, Baylin SB and Sidransky D (1994) Homozygous deletion on chromosome 9p and loss of heterozygosity on 9q, 6p, and 6q in primary human small cell lung cancer. *Cancer Res* **54**: 2322–2326

Miura K, Suzuki K, Tokino T, Isomura M, Inazawa J, Matsuno S and Nakamura Y (1996) Detailed deletion mapping in squamous cell carcinoma of the esophagus narrows a region containing a putative tumor suppressor gene to about 200 kilobases on distal chromosome 9q. *Cancer Res* **56**: 1629–1634

Nobori T, Miura K, Wu DJ, Lois A, Takabayashi K and Carson DA (1994) Deletions of the cyclin-dependent kinase-4 inhibitor gene in multiple human cancers. *Nature* **368**: 753–756

Orlow I, Lianes P, Lacombe L, Dalbagni G, Reuter VE and Cordon-Cardo C (1994) Chromosome 9 allelic losses and microsatellite alterations in human bladder tumors. *Cancer Res* **54**: 2848–2851

Polascik TJ, Cairns P, Chang WYH, Schoenberg MP and Sidransky D (1995) Distinct regions of allelic loss on chromosome 4 in human primary bladder carcinoma. *Cancer Res* **55**: 5396–5399

Ruppert JM, Tokino K and Sidransky D (1993) Evidence for two bladder cancer suppressor loci on human chromosome 9. *Cancer Res* **53**: 5093–5095

Sambrook J, Fritsch E and Maniatis T (1989) *Molecular Cloning: a Laboratory Manual*. Cold Spring Harbor Laboratory: New York

Shanley SM, Dawkins H, Wainwright BJ, Wicking C, Heenan P, Eldon M, Searle J and Chenevix-Trench G (1995) Fine deletion mapping on the long arm of chromosome 9 in sporadic and familial basal cell carcinomas. *Hum Mol Genet* **4**: 129–133

Shipman R, Schrami P, Colombi M, Raefle G and Ludwig CU (1993) Loss of heterozygosity on chromosome 11p13 in primary bladder carcinoma. *Hum Genet*, **91**: 455–458

Shultz DC, Vanderveer L, Buetow KH, Boente MP, Ozols RF, Hamilto TC and Godwin AK (1995) Characterization of chromosome 9 in human ovarian neoplasia identifies frequent genetic imbalance on 9q and rare alterations involving 9p, including *CDKN2*. Cancer Res **55**: 2150–2157.

Simoneau AR, Spruck CH Jr, Gonzalez-Zulueta M, Gonzalgo ML, Chan MF, Tsai YC, Dean M, Steven K, Horn T and Jones PA (1996) Evidence for two tumor suppressor loci associated with proximal chromosome 9p to q and distal chromosome 9q in bladder cancer and the initial screening for GAS1 and PTC mutations. *Cancer Res* **56**: 5039–5043

Southgate J, Proffitt J, Roberts P, Smith B and Selby P (1995) Loss of cyclin-dependent kinase inhibitor genes and chromosome 9 karyotypic abnormalities in human bladder cancer cell lines. *Br J Cancer* **72**: 1214–1218

Takle LA and Knowles MA (1996) Deletion mapping implicates two tumour suppressor genes on chromosome 8p in the development of bladder cancer. *Oncogene* **12**: 1083–1087

Tamimi Y, Bringuier PP, Smit F, van Bokhoven A, Abbas A, Debruyne FM and Schalken JA (1996) Homozygous deletions of p16(INK4) occur frequently in bilharziasis-associated bladder cancer. *Int J Cancer* **68**: 183–187

Van Tilborg AAG, Hekman ACP, Vissers KJ, van der Kwast TH and Zwarthoff EC (1998) Loss of heterozygosity on chromosome 9 and loss of chromosome 9 copy number are separate events in the pathogenesis of transitional cell carcinoma of the bladder. *Int J Cancer* **75**: 9–14

Williamson MP, Elder PA, Shaw ME, Devlin J and Knowles MA (1995) p16 (*CDKN2*) is a major deletion target at 9p21 in bladder cancer. *Hum Mol Genet* **4**: 1569–1577

*© Cancer Research Campaign 1999*
